# HIV-1 exploits importin 7 to maximize nuclear import of its DNA genome

**DOI:** 10.1186/1742-4690-6-11

**Published:** 2009-02-04

**Authors:** Lyubov Zaitseva, Peter Cherepanov, Lada Leyens, Sam J Wilson, Jane Rasaiyaah, Ariberto Fassati

**Affiliations:** 1 Wohl Virion Centre, Division of Infection and Immunity, University College London (UCL), London, UK; 2 MRC Centre for Medical Molecular Virology, Division of Infection and Immunity, University College London (UCL), London, UK; 3 Division of Medicine, St Mary's Campus, Imperial College London, Norfolk Place, London, W2 1PG, UK; 4 Centre for Post-genomic Virology, Division of Infection and Immunity, University College London, 46 Cleveland Street, London, W1T 4JF, UK

## Abstract

**Background:**

Nuclear import of the HIV-1 reverse transcription complex (RTC) is critical for infection of non dividing cells, and importin 7 (imp7) has been implicated in this process. To further characterize the function of imp7 in HIV-1 replication we generated cell lines stably depleted for imp7 and used them in conjunction with infection, cellular fractionation and pull-down assays.

**Results:**

Imp7 depletion impaired HIV-1 infection but did not significantly affect HIV-2, simian immunodeficiency virus (SIVmac), or equine infectious anemia virus (EIAV). The lentiviral dependence on imp7 closely correlated with binding of the respective integrase proteins to imp7. HIV-1 RTC associated with nuclei of infected cells with remarkable speed and knock down of imp7 reduced HIV-1 DNA nuclear accumulation, delaying infection. Using an HIV-1 mutant deficient for reverse transcription, we found that viral RNA accumulated within nuclei of infected cells, indicating that reverse transcription is not absolutely required for nuclear import. Depletion of imp7 impacted on HIV-1 DNA but not RNA nuclear import and also inhibited DNA transfection efficiency.

**Conclusion:**

Although imp7 may not be essential for HIV-1 infection, our results suggest that imp7 facilitates nuclear trafficking of DNA and that HIV-1 exploits imp7 to maximize nuclear import of its DNA genome. Lentiviruses other than HIV-1 may have evolved to use alternative nuclear import receptors to the same end.

## Background

Akin to other lentiviruses, HIV-1 is able to infect primary non-dividing cells, such as tissue macrophages, microglial cells and CD4+ memory T-cells as well as cells artificially arrested in the cell cycle (reviewed in reference [1]). These primary cells represent key in vivo targets for virus transmission and AIDS pathogenesis, hence the importance of understanding how HIV-1 can traverse the intact nuclear envelope.

Biochemical studies have shown that the ability of HIV-1 to infect non-dividing cells depends on the active nuclear import of its intracellular reverse transcription/pre-integration complex (RTC/PIC) [2], and both viral and cellular elements have been implicated in this process [1,3]. Chimeric viruses, in which HIV-1 Gag has been replaced with Moloney murine leukaemia (MLV) Gag, infect cell cycle-arrested cells with very low efficiency [4]. Within MLV Gag, the CA protein appears to be the dominant negative regulator of nuclear import [4]. MLV can only infect cycling cells [5-7], and its RTC retains a significant portion of CA protein until after nuclear entry [8]. Moreover, HIV-1 mutants that do not shed enough p24 CA are defective in nuclear import and integration [9]. Based on this evidence, it has been proposed that appropriate shedding of CA protein from the RTC/PIC is a key step for nuclear import of HIV-1 [1,10]. HIV-1 capsid exceeds the maximal functional diameter of the nuclear pore complex, hence it is likely that uncoating takes place before RTCs can be imported into nuclei.

Additional viral elements implicated in HIV-1 nuclear import include p17 MA, Vpr, integrase (IN), and the central polypurine tract (cPPT) [1,3]. The cPPT is a second origin of DNA plus strand synthesis located within *pol *that, after completion of reverse transcription, results in a short (approximately 100 nt) stretch of triple stranded DNA [11]. When included in HIV-1 vectors, the cPPT increases nuclear accumulation of vector DNA 2- to 10-fold (reviewed in reference [1]), although there is controversy on the magnitude of its effect in the context of wild-type virus replication [12-16]. Recent data suggest that formation of the triple stranded DNA stretch promotes nuclear transport of HIV-1 PICs by inducing timely uncoating of the viral capsid [17].

Following uncoating, the HIV-1 RTC, containing viral nucleic acids as well as cellular and viral components, must engage one or more cellular pathways for nuclear import. Several cellular factors have been shown to participate in the trafficking RTCs to the nucleus, including fasciculation and elongation protein zeta-1 (FEZ1), Nup98, Nup358, Nup153, importin 7 (imp7), and transportin 3/transportin SR-2 (tnp3) [18-23]. Furthermore, recent evidence indicated that HIV-1 can recruit a newly discovered cellular pathway for retrograde transport of tRNAs in mammalian cells to promote RTC nuclear import [24,25].

Imp7 is a nucleocytoplasmic transport protein closely related to impβ and its N-terminus binds to RanGTP [26]. Imp7 serves as an import factor for some ribosomal proteins, the glucocorticoid receptor, zinc finger protein EZI, ERK MAP kinase, activated Smads, and, as a heterodimer with impβ, histone H1 [27-32]. Moreover, nuclear import of the adenoviral DNA-binding protein pVII and of HIV-1 integrase (IN) and Rev is supported by imp7 and other importins, whereas adenovirus type 2 nuclear import depends on the imp7/impβ heterodimer [20,33-35].

Using in vitro nuclear import assays, Imp7 was shown to promote nuclear import of HIV-1 RTCs in a Ran- and energy-dependent manner. Furthermore, transient knock down of imp7 by siRNA inhibited HIV-1 infection [20], although the latter observation has been questioned [36]. Double imp7 knock down in both viral producer cells and target cells was required for maximal inhibition of viral infection [37]. Imp7 was shown to bind to the C-terminal region of HIV-1 IN, and mutant viruses unable to interact with imp7 were defective in both reverse transcription and nuclear entry [37].

To gain a better understanding of HIV-1 nuclear trafficking, we established cells stably knocked down for imp7 and performed infection and biochemical fractionation assays. Our results indicate that HIV-1, but not HIV-2, SIVmac or EIAV, recruits imp7 via interaction with IN to maximise viral DNA nuclear import. Our data argue that the closely related lentiviruses may have evolved different and probably redundant nuclear import mechanisms.

## Results

**Figure 1:**
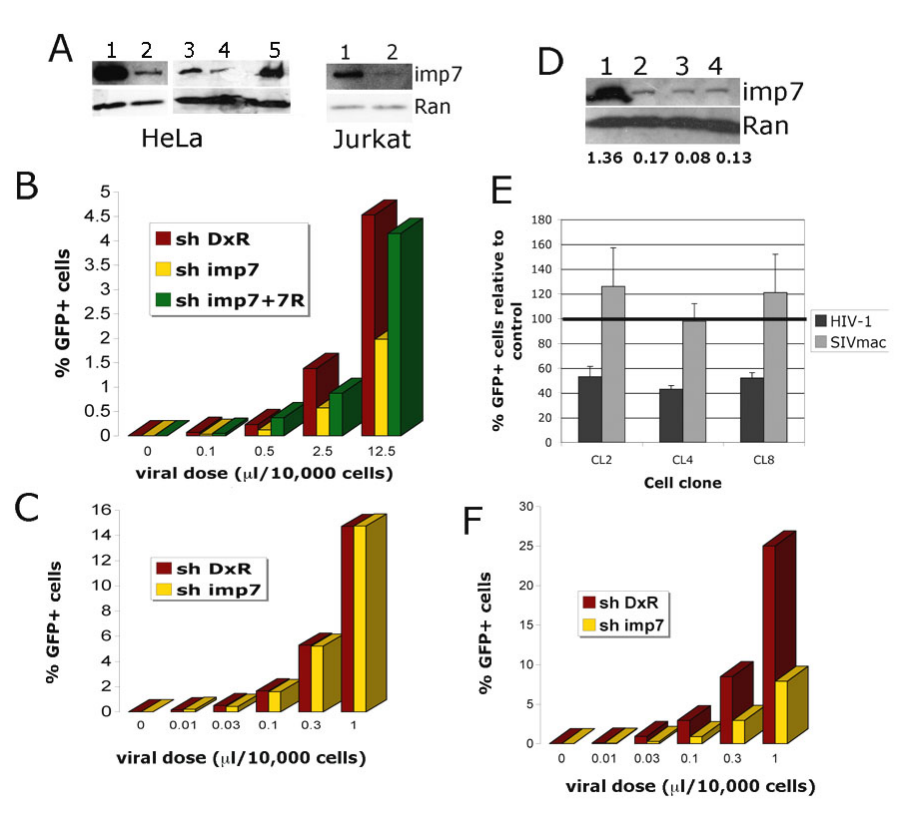
**Generation of stable imp7 knock down (KD) cells**. (A) Left panels: Western blot with an anti-imp7 antibody and an anti-Ran antibody on total cell extracts obtained from a low passage polyclonal population of DxR KD (lane 1) and imp7 KD HeLa cells (lane 2), a polyclonal population of DxR KD (lane 3), imp7KD (lane 4) and the same imp7 KD population expressing an imp7 cDNA with two silent mutations making it resistant to the shRNA (imp7+7R) (lane 5). Note that cells in lanes 3 to 5 were grown for 4 weeks to allow for selection of the imp7+7R line. Right panel: Western blot with an anti-imp7 antibody and an anti-Ran antibody on total cell extracts obtained from a polyclonal population of DxR KD cells (lane 1) and imp7 KD Jurkat cells (lane 2). (B) DxR KD (shDxR), imp7 KD (shimp7) and imp7 back complemented (shimp7+7R) HeLa cells were plated onto 24-well plates to have the same confluency by the next day and infected with five fold serial dilutions of a VSV-G pseudotyped HIV-1 vector (pHR') expressing GFP. Twenty-four hours after infection the percentage of GFP+ cells was measured by flow cytometry. (C) Cells were infected with three fold serial dilutions of a VSV-G pseudotyped SIVmac vector expressing GFP and analysed as in (B). Similar results were obtained in least three independent experiments using different virus stocks. (D) Western blot with an anti-imp7 antibody and an anti-Ran antibody on total cell extracts obtained from a polyclonal population of DxR KD HeLa cells (lane 1) and three different imp7 KD clones (lane 2, clone 2; lane 3, clone 4; lane 4, clone 8). The bands were scanned and the intensity of the imp7 band relative to Ran is shown below each sample. (E) DxR KD cells and imp7 KD clonal cell populations were infected with a VSV-G pseudotyped HIV-1 vector (pHR') or SIVmac vector expressing GFP at an MOI of 0.03 and the percentage of GFP+ cells counted 24 hours after infection by flow cytometry. Data are expressed as average percentage of infection relative the parent line control (shDxR) ± SD of three independent experiments. (F) DxR KD and imp7 KD Jurkat cells were infected with three fold serial dilutions of a VSV-G pseudotyped HIV-1 vector (pCSGW) expressing GFP and the percentage of GFP+ cells counted 24 hours after infection by flow cytometry. Similar results were obtained in two additional experiments using different virus stocks.

To investigate the function of imp7 in HIV-1 infection, HeLa polyclonal cell lines with a stable imp7 knock down (KD) were generated by shRNA expressed from a lentiviral vector [38]. Control cells were similarly generated that harboured a shRNA targeting the *Discosoma corallimorpharian *DsRed mRNA (DxR). The control shRNA effectively knocked down expression of the DxR protein in control experiments, indicating that it was indeed recruited into the RISC complex and was functional (not shown). As an additional control for specificity, polyclonal imp7 KD cells were back complemented with an imp7 cDNA harbouring two silent mutations making it resistant to the imp7 shRNA. Cells were analyzed by Western blotting with antibodies against imp7 and Ran (as a loading control). Imp7 KD in HeLa cells was robust and specific and back complementation with the mutant cDNA effectively restored imp7 expression. Effective imp7 KD was also achieved in human lymphocytic Jurkat cells (Figure 1A).

Imp7 KD cells grew slower than control DxR KD cells and back complemented cells, consistent with our previous findings using transient imp7 KD in human cells [20]. Importantly, however, cell viability was not significantly affected by stable depletion of imp7 (Additional file 1 and Table 1). Profiling of the cell cycle with propidium iodide showed minor differences between imp7 KD and control cells with a trend towards a reduction of the percentage of cells in G1 and an increase in S (HeLa) and G2/M phases (Jurkat) (Table 1). These results suggested that imp7 KD modestly delayed cell cycle progression.

**Table 1:** Effects of imp7 KD on cell viability and cell cycle progression

Cell type	% Live	% Dead	% G1	% S	% G2/M
HeLa DxR	98.81	1.2	59.39	16.12	21.29
HeLa CL2	97.69	2.31	57.37	17.91	18.7
HeLa CL4	98.72	1.28	56.11	18.29	21.4
HeLa7+7R	99.4	0.6	59.86	16.2	19.07
Jurkat DxR	89.21	10.79	56.8	13.42	16.64
Jurkat sh7	86.71	13.29	50.44	12.92	20.17

Cells were analyzed by flow cytometry following differential fluorescent staining of live and dead cells and propidium iodide staining to examine cell cycle.

The imp7 KD cells were infected with different doses of Vesicular Stomatitis glycoprotein G (VSV-G) pseudotyped HIV-1 vectors expressing green fluorescent protein (GFP) [39]. The results in Figure 1B show that imp7 KD resulted in a specific inhibition of HIV-1 vector infection. Similar results were also obtained with a different HIV-1 derived vector [40], as well as with a full length HIV-1_LAI_Δenv virus that expressed GFP in place of Nef (not shown) [4]. Intriguingly, when challenged with a simian immunodeficiency virus (SIVmac) vector [41] imp7 and DxR KD cells were equally infected (Figure 1C). HIV-2 infection was very modestly impaired in imp7 KD cells (not shown). These unexpected results indicated that cell toxicity or other non-specific side effects could not explain the lower levels of HIV-1 infection observed in imp7 KD cells. Several imp7 KD HeLa clones were examined. Imp7 protein levels in these clones ranged from approximately 15% to 6% of those in the parental cell line (Figure 1D), and a correlation between reduction in HIV-1 infection and levels of imp7 KD was apparent (Figure 1E). The efficiency of HIV-1 vector infection was also lower in imp7 KD compared to control DxR KD Jurkat cells (Figure 1F). Taken together, these results indicated that HIV-1 infection was specifically impaired in imp7 KD cells.

Inhibition of HIV-1 infection in imp7 KD cells was maximal between MOIs of 0.01 and 0.06 and was partially overcome at an MOI ≈ 1 (Additional file 2). To examine if imp7 in the producer cells was important, virus was produced in imp7 KD or DxR cells, normalized for RT activity and used to infect imp7 KD or DxR target cells at an MOI of 0.05. In agreement with a previous report [37], the virus produced in imp7 KD cells had a lower relative infectivity compared to virus produced in DxR cells. However the difference in infectivity between the two viruses did not reach statistical significance in imp7 KD target cells (Additional file 2).

### Cell cycle arrest does not influence HIV-1 infection efficiency in imp7 KD cells

**Figure 2:**
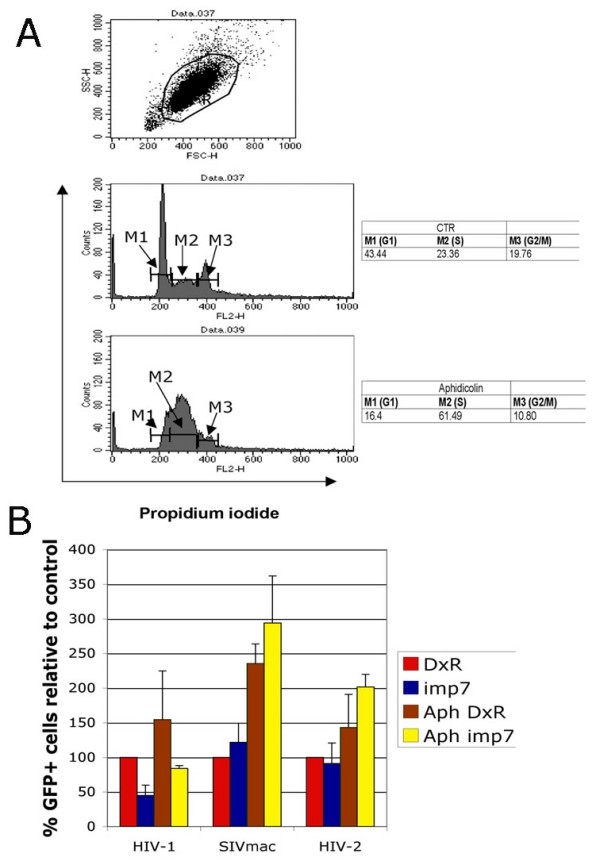
**The effect of imp7 KD on HIV-1 infection is independent of cell-cycle arrest**. (A) HeLa cells analyzed by FACS after staining with propidium iodide. Top panel, dot plot of forward and side scatter; the gated population was used for cell cycle analysis, middle panel, no aphidicolin, bottom panel, cells treated with 1.5 micrograms/ml aphidicolin. M1, G1 phase; M2, S phase; M3, G2/M phase. (B) FACS analyses of HeLa DxR and imp7 KD cells infected at an MOI of 0.03 with a VSV-G pseudotyped HIV-1 vector (pCSGW), SIVmac vector and HIV-2 vector in the absence or presence (Aph) of aphidicolin. Cells were analyzed for GFP expression 20–24 hours post infection. Bars represent the mean value ± SD of three independent experiments. Infection levels with an MLV vector were 10 fold lower in aphidicolin-treated cells compared to control cells (not shown).

Imp7 may be required to promote RTC/PIC nuclear translocation only when the nuclear envelope is present. Hence in the experiments shown in Figure 1, HIV-1 might have accessed the nucleus in mitotic cells when the nuclear envelope was dissolved, independently from imp7. To test this possibility, cells were treated with aphidicolin to block progression of the S-phase. Flow cytometry analysis confirmed that exposure to the drug inhibited accumulation of cells in the S-phase (Figure 2A). Cell-cycle arrest increased susceptibility of the cells to infection with HIV-1, HIV-2 and SIVmac (Figure 2B). Importantly, the relative difference in HIV-1 infection efficiency between DxR and imp7 KD cells observed in cycling cells was maintained following cell cycle arrest (Figure 2B). In contrast, infectivity of SIVmac and HIV-2 were not affected in imp7 KD cells in either condition (Figure 2B).

### The binding affinity of different lentiviral INs for imp7 correlates with infection phenotype in imp7 KD cells

**Figure 3:**
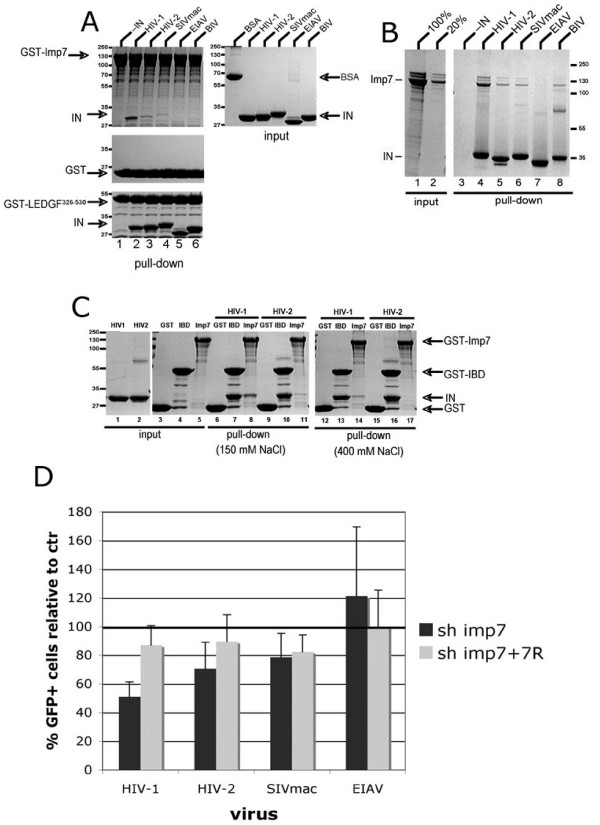
**Lentiviral IN affinity for imp7 correlates with infection phenotype in imp7 KD cells**. (A) GST-imp7 (top left), GST (middle), or GST-LEDGF^326–530 ^(bottom) immobilized on glutathione sepharose beads were incubated in the absence (lane 1), or presence (lanes 2–6) of untagged recombinant HIV-1, HIV-2, SIVmac, EIAV, or BIV IN in the pull-down buffer containing 130 mM NaCl. Proteins bound to glutathione sepharose beads were resolved in an SDS PAGE gel and detected by staining with Coomassie Blue. Input quantities of each soluble protein used are shown to the right. Migration positions of GST-Imp7, INs, GST, and GST-LEDGF^326–530^, and the molecular weight markers are indicated. (B) Non-tagged Imp7 was incubated in the absence (lane 3) or presence (lanes 4–9) of C-terminally hexahistidine-tagged INs from HIV-1, HIV-2, SIVmac, EIAV, BIV and Ni-NTA agarose beads in a pull down buffer containing 150 mM NaCl. Proteins captured on the resin were separated in a tricine SDS PAGE gel and detected with Coomassie Blue. Lanes 1 and 2 show 100% and 20% Imp7 input, respectively. (C) GST (lanes 3, 6, 9, 12, 15), GST-LEDGF^326–530 ^(lanes 4, 7, 10, 13, 16), or GST-imp7 (lanes 5, 8, 11, 14, 17) were incubated without (lanes 3–5), or with non-tagged HIV-1 (lanes 6–8, 12–14) or HIV-2 (lanes 9–11, 15–16) INs. The pull-down buffer contained 150 mM (lanes 3–11) or 400 mM (lanes 12–17) NaCl. Lanes 1 and 2 contained input quantities of HIV-1 and HIV-2 INs, respectively. (D) DxR KD and imp7 KD cell populations were infected with VSV-G pseudotyped HIV-1, (pHR'), SIVmac, HIV-2 and EIAV vectors expressing GFP at an MOI of 0.03 and the percentage of GFP+ cells counted 24 hours after infection by flow cytometry. Data are expressed as average percentage of infection relative to control (shDxR) ± SD of two independent experiments performed in duplicate.

The finding that SIVmac and HIV-2 infection was not impaired in imp7 KD cells was unexpected and we sought to understand the reason for this difference with HIV-1. Imp7 was shown to bind HIV-1 IN [20,37], and the mutations affecting this interaction resulted in viruses defective for reverse transcription and nuclear import [37], supporting its functional relevance. Hence several lentiviral INs were examined in pull down assays to test their ability to interact with imp7. GST-imp7 was immobilized on glutathione sepharose beads and the IN proteins from HIV-1, HIV-2, SIVmac, EIAV, and bovine immunodeficiency virus (BIV) were tested. GST-LEDGF^326–530^, containing the IN binding domain (IBD) of LEDGF/p75 [42], the host factor displaying broad affinity for INs of lentiviral origin [43-46], and GST were used as positive and negative controls, respectively. In agreement with previously reported results [20,37] GST-Imp7 efficiently pulled down HIV-1 IN (Figure 3A). In contrast, although copious amounts of all lentiviral INs were recovered with GST-LEDGF^326–530^, the INs from HIV-2, SIVmac, and BIV only weakly interacted with Imp7, and no detectable interaction was observed for EIAV IN (Figure 3A). Importantly, none of the IN proteins were recovered with GST- loaded resin, confirming specificity of the observed interactions (Figure 3A). Essentially identical results were obtained in a reverse pull down experiment using hexahistidine-tagged INs immobilized on Ni-NTA agarose beads and untagged Imp7 (Figure 3B). The interaction between nuclear import receptors and their cargo are generally mediated by charge-charge interactions [28]. Concordantly, the imp7-IN interaction was sensitive to the ionic strength of the pull down buffer (Figure 3C). However, while in the presence of 400 mM NaCl the interaction of imp7 with HIV-1 IN was still detectable, pull down of HIV-2 IN was abolished (Figure 3C). Overall, the infection phenotype of different lentiviruses in imp7 KD cells correlated well with the affinity of their respective INs for imp7 as detected in the pull down experiments (Figures 2B and 3D). These results strongly support the functional relevance of HIV-1 IN interaction with imp7 and explain why lentiviruses other than HIV-1 are not impaired in imp7 KD cells.

### Efficient accumulation of HIV-1 DNA in nuclear fractions of infected cells

**Figure 4:**
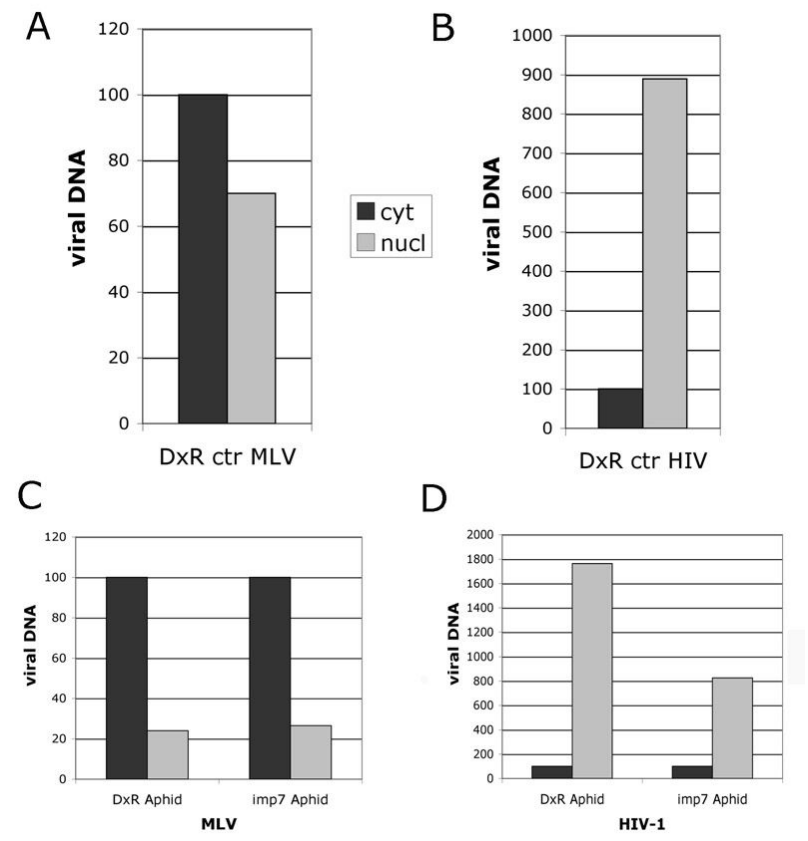
**Rapid and efficient nuclear accumulation of HIV-1 DNA**. Cells were plated in 10 cm plates, co-infected with a HIV-1 and a MLV vector at an MOI of approximately 0.1, incubated at 4°C for 2 hours and then at 37°C for an additional 24 hours. Cytoplasm and nuclei were fractionated and the distribution of MLV vector DNA (A) and HIV-1 vector DNA (B) was examined by Taqman PCR in the same HeLa DxR cells. DxR and imp7 KD cells were co-infected with a MLV and a HIV-1 vector after treatment with 1.5 micrograms/ml aphidicolin for 24 hours. The distribution of MLV vector DNA (C) and HIV-1 vector DNA (D) was examined by Taqman PCR. Bars represent the mean value of two independent experiments. To normalise across experiments, the total amount of viral DNA detected in the cytoplasm was given an arbitrary value of 100.

To test if nuclear import was the actual step inhibited in imp7 KD cells, untreated or cell cycle-arrested cells were co-infected with MLV and HIV-1 vectors [47]. Twenty-four hours post infection, cells were subjected to fractionation to separate nuclei from cytoplasm [48] (see also Figure 6), and the distribution of MLV and HIV-1 DNA examined using Taqman real time PCR. No "cross amplification" was observed in independent reactions. MLV DNA was distributed rather evenly in the nuclear and cytoplasmic fractions of normal cells and was less abundant in the nuclear fraction of cell cycle-arrested cells (Figures 4A and 4C). In contrast, HIV-1 DNA was found predominantly in the nuclear fractions of both untreated and cell cycle-arrested cells (Figures 4B and 4D). In fact, cell cycle-arrested cells contained more HIV-1 DNA associated with the nuclei than control cells, in agreement with the higher infectivity observed following cell cycle arrest (Figure 2B). However, considerably lower levels of HIV-1 DNA were found in the nuclear fraction of imp7 KD cells compared to DxR cells (Figure 4), in agreement with the infection levels (Figure 2B). Taken together these results suggested that imp7 KD inhibited HIV-1 accumulation at or within the nuclei.

### Imp7 influences the degree of HIV-1 nuclear import

**Figure 5:**
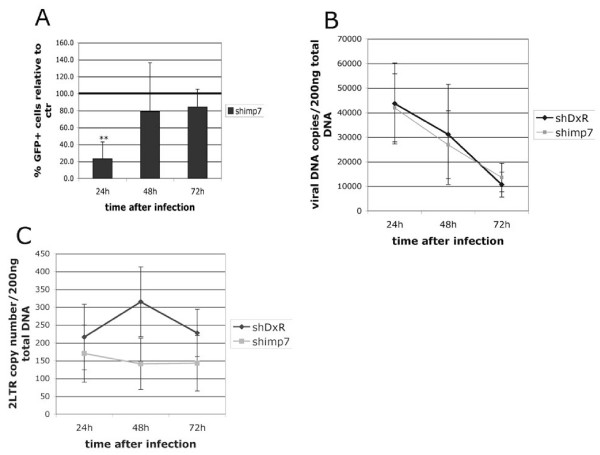
**Imp7 KD impacts on the efficiency of HIV-1 nuclear import**. DxR and imp7 KD HeLa cells were infected with an HIV-1 vector (pCSGW) at an MOI of approximately 0.1 and analyzed at the indicated time points by (A) flow cytometry to measure GFP+ cells, (B) Taqman PCR to measure total viral DNA copy number, (C) Taqman PCR to measure 2LTR circular DNA copy number. Data are expressed as mean value ± SD of three independent experiments. Statistical significance was calculated by the Student t-test (n = 3), ** p < 0.005.

The data shown in Figure 4B indicated that trafficking of HIV-1 to the nucleus is remarkably efficient. To test if imp7 KD influenced this process, a time course experiment was carried out. Imp7 KD and DxR cells were infected with an HIV-1 vector expressing GFP and the percentage of GFP+ cells, the amount of total and 2LTR circular viral DNA were measured 24 h, 48 h and 72 h post infection (we were unable to detect 2LTR circular viral DNA earlier than 24 h post-infection). HIV-1 infection efficiency was significantly lower in imp7 KD than in DxR cells at 24 h (Student t-test p < 0.005, n = 3), but was similar at 48 h and 72 h post-infection (Figure 5A). Viral DNA synthesis was equal in the two cell types (Figure 5B). Total viral DNA steadily decreased from 24 h to 72 h post-infection at the same rate in both cell types, presumably due to degradation and dilution of un-integrated viral DNA. 2LTR circular viral DNA, a hallmark of nuclear entry, was consistently higher in DxR than in imp7 KD cells, with the widest gap at 48 h, the difference being statistically significant (Student t-test, p < 0.03, n = 3) (Figure 5C). These data suggested that imp7 KD perturbs HIV-1 DNA nuclear import.

### Imp7 KD inhibits HIV-1 DNA but not RNA nuclear import

DNA can be imported into the nucleus of non-dividing cells [49,50], and imp7 has been shown to promote DNA-containing HIV-1 RTCs nuclear import in an in vitro assay [20]. Hence we hypothesized that HIV-1 RTCs and perhaps DNA, in general, may both exploit imp7 to promote their nuclear entry. Reverse transcription of the HIV-1 genome into a DNA molecule would then be a requirement for nuclear import.

**Figure 6:**
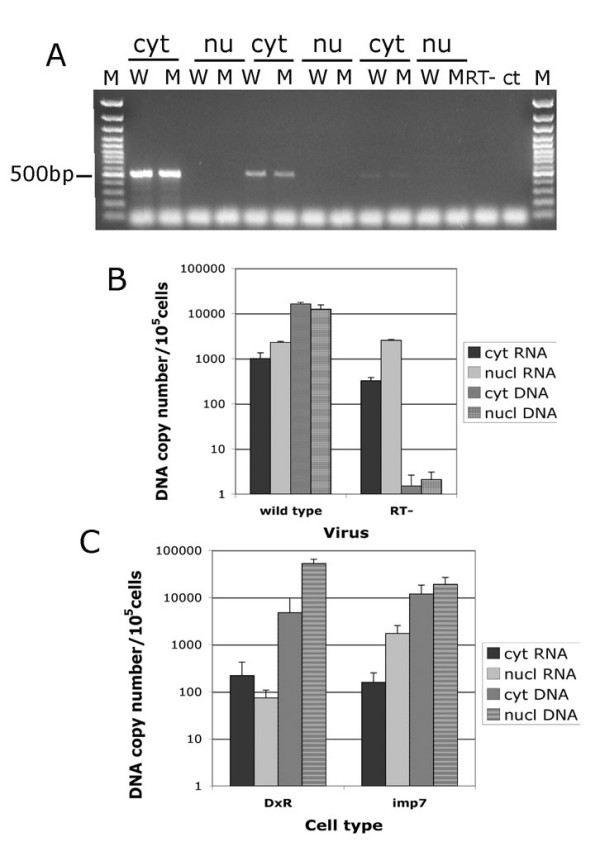
**Imp7 KD inhibits viral DNA but not viral RNA accumulation in the nuclei**. (A) HeLa cells were infected with a DNAse-treated and purified HIV-1 vector and incubated for 2 hours at 4°C and then 4–6 hours at 37°C. Infected cells were fractionated into nuclear and (a) cytoplasmic fractions and nucleic acids extracted, purified and divided into two aliquots. One aliquot was treated with RNAse A, re-purified and used for DNA quantification. The other aliquot was treated with RNAse-free DNAse and used for first-strand cDNA synthesis. Cyclophillin A cDNA was then amplified by PCR in each fraction to examine cross-contamination of nuclear fractions with cytoplasmic material and the overall efficiency of first-strand cDNA synthesis. Cyt, cytoplasmic fractions; Nu, nuclear fractions; W, wild-type virus; M, mutant virus; RT-, cytoplasmic fraction with no RT during first-strand cDNA synthesis; ctr-, primers only; Mw, GeneRuler 100 bp DNA molecular weigh marker. The band migrating at approximately 500 bp is cyclophilin A, lower molecular weigh bands are PCR artefacts. The experiments were performed using 10 fold serial dilutions of the cDNA mix. (B) HIV-1 RNA accumulates in the nuclei shortly after infection. HeLa cells were infected at an MOI of approximately 0.2 with a VSV-G pseudotyped HIV-1 vector (wild type) or with the same amount (p24 normalized) of vector with a mutation in RT and unable to reverse transcribe (RT-). Cells were incubated for 2 hours at 4°C and then 4 hours at 37°C following which samples were fractionated in nuclear and cytoplasmic fractions and treated as described in (A). Taqman PCR was used to measure the amount of viral DNA and RNA in each fraction. First-strand cDNA synthesis reactions carried out in the absence of RT gave undetectable signal. Values shown are average values ± SD of triplicate experiments. Similar results were obtained in two independent experiments. (C) Accumulation of HIV-1 DNA is reduced in imp7 KD cells. HeLa DxR or imp7 KD cells were infected with the same dose of a VSV-G pseudotyped HIV-1 vector, incubated 2 hours at 4°C and then 6 hours at 37°C, following which nuclear and cytoplasmic extracts were prepared and treated as described in (A). After first-strand cDNA synthesis, Taqman PCR was used to measure the amount of viral DNA and RNA in each fraction. First-strand cDNA synthesis reactions carried out in the absence of RT gave undetectable signal. Values shown are average copy number of viral RNA or DNA/μg total nucleic acids ± SD of triplicate experiments. Similar results were obtained in two independent experiments.

To test this hypothesis, we infected HeLa cells with equal amounts (p24-normalised) of a wild type or reverse transcriptase (RT)-deficient HIV-1 vector. The latter carries an inactivating mutation within the highly conserved YMD^185^D motif of the RT catalytic site and, hence, is unable to reverse transcribe [51,52]. The mutant virus displays no assembly, release or post-entry defects [38]. Four hours post infection nucleic acids isolated from cytoplasmic and nuclear fractions were divided into two aliquots: one aliquot was treated with RNAseA and used for DNA quantification; the other aliquot was first digested with RNAse-free DNAse and then used for first strand cDNA synthesis. To check the effectiveness of the fractionation procedure, the distribution of the spliced cyclophilin A mRNA was examined by RT-PCR. Because cyclophilin A mRNA is abundant [53], it served as an excellent control for possible contamination of the nuclear fractions with cytoplasmic material and as a internal standard for the quality/quantity of cDNA synthesis. Figure 6A shows that cytoplasmic contamination of the nuclear fraction was less than 1%. Results shown in Figure 6A also indicated that anything detected in the nuclear fraction was most likely inside the nuclei and not simply associated with the external nuclear envelope. The external nuclear membrane is a functional part of the rough endoplasmic reticulum (ER) [54], hence its incomplete dissociation should result in detection of ribosome-bound cyclophilin A mRNA in the nuclear fraction.

The distribution of HIV-1 RNA and DNA was examined by Taqman real time PCR using primers specific for the vector sequence. Intriguingly, viral RNA was readily detected in the nuclei of acutely infected cells (Figure 6B). This was not due to small amounts of synthesized DNA present in the RTCs because viral RNA was found in the nuclei of cells infected with the mutant vector unable to reverse transcribe (Figure 6B). Therefore, viral DNA synthesis does not appear to be absolutely required for nuclear import of the HIV-1 RTC.

Next, we tested if imp7 KD influenced the distribution of viral DNA and RNA in acutely infected cells. The same fractionation procedure was used, except that imp7 KD and DxR KD cells were contrasted and infection was carried on for six hours to allow more time for the build up of possible differences. These experiments confirmed that viral RNA could be detected within the nuclei shortly after infection. However, viral DNA accumulated more efficiently in the nuclei of DxR cells than in those of imp7 KD cells, and conversely viral RNA accumulated less efficiently in the nuclei of DxR KD cells than in those of imp7 KD cells (Figure 6C), suggesting that DNA and RNA nuclear import may compete for access or passage through nuclear pore complexes. Of note, in all experiments omission of RT during first strand cDNA synthesis resulted in undetectable signals (not shown). Taken together, these results suggested that imp7 KD selectively inhibited viral DNA nuclear import.

### Imp7 KD reduces the efficiency of plasmid DNA transfection

**Figure 7:**
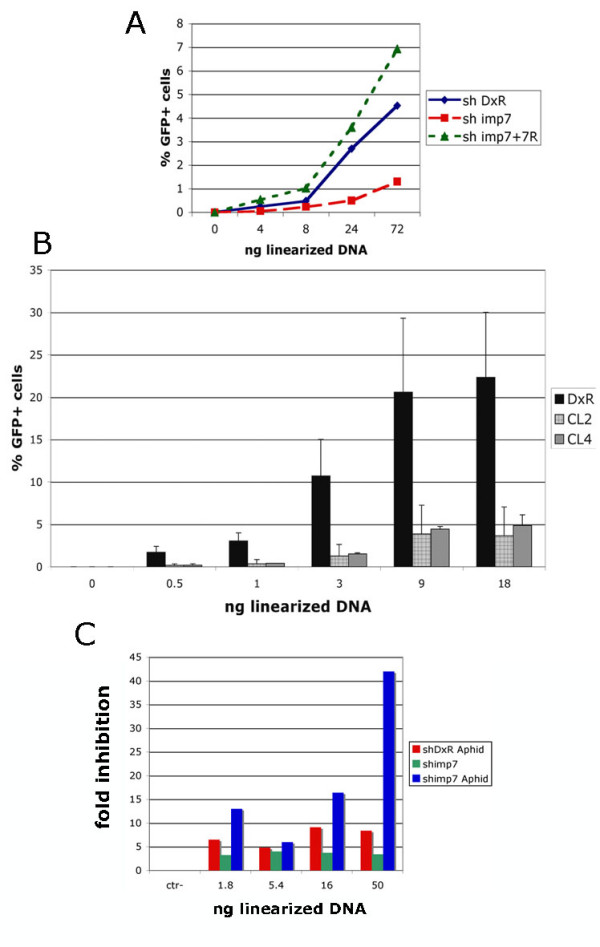
**Imp7 KD inhibits plasmid DNA transfection**. (A) HeLa DxR KD (shDxR), imp7 KD (shimp7) and imp7 back complemented cells (shimp7+7R) were plated onto 6-well plates and transfected the next day with different amounts of the linearized HIV-1 plasmid DNA (pHR') complexed with FuGene 6. Twenty-four hours after transfection, the percentage of GFP+ cells was measured by flow cytometry. (B) HeLa DxR KD cells (DxR) and two different imp7 KD clones (please refer to Figure 2D) were plated onto 24-well plates and transfected as described in panel A. GFP+ cells were counted twenty four hours after transfection by flow cytometry. Note that 1/4 of the DNA was used in experiments shown in panel B compared to panel A because cells were plated onto 24-well plates. Bars represent the average value ± SD of three independent experiments. (C) HeLa DxR and imp7 KD cells were plated into 6 well plates with or without 1.5 micrograms/ml aphidicolin for 24 hours. Cells were then transfected with the indicated amount of linearized plasmid DNA (pHR') as described in (A) and analyzed by flow cytometry 24 hours later. Data are expressed as fold inhibition relative to shDxR cells and are representative of two independent experiments.

Results shown in Figure 6 suggested that imp7 KD selectively inhibited nuclear import of HIV-1 DNA. To test if imp7 could promote nuclear trafficking of other forms of DNA, the efficiency of plasmid DNA transfection in imp7 and DxR KD cells was examined. To mimic more closely the situation with the RTC/PIC, the HIV-1 vector plasmid DNA was linearized by restriction enzyme digestion. As shown in Figure 7, the efficiency of DNA transfection (measured as the percentage of GFP+ cells) was significantly lower in imp7 KD cells than in DxR KD cells and this phenotype was reversed upon imp7 back complementation. Similar results were obtained in two different imp7 KD clones (Figure 7B). Moreover, the inhibition of DNA transfection was observed using the SIVmac vector plasmid and a non-retroviral plasmid (not shown), indicating that it was not DNA sequence-dependent.

Imp7 KD cells grew more slowly than DxR cells and this might have influenced DNA transfection efficiency. To rule out this possibility, imp7 KD and DxR cells were arrested in the cell cycle by treatment with aphidicolin so that conditions for both cell types were equal at the time of transfection. Treated and untreated cells were transfected with linearized plasmid DNA and analyzed for GFP expression 24 hours later (Figure 7C). Cell cycle arrest clearly reduced the efficiency of DNA transfection in all cell types but the reduction was significantly more pronounced in imp7 KD cells compared to DxR cells. Similar results were obtained with circular plasmid DNA (not shown). These data suggested that differences in the rate of cell division could not account for the differences in DNA transfection efficiency observed in DxR and imp7 KD cells.

## Discussion

We have found that HIV-1 DNA associates with nuclei of infected cells with remarkable efficiency and speed, that imp7 influences the kinetics of HIV-1 DNA nuclear trafficking, and that interaction of imp7 with IN is critical to this end. Furthermore, our data suggest that lentiviruses may have evolved different mechanisms for nuclear trafficking of their RTCs.

Several lines of evidence support our conclusions. HIV-1 infection was impaired in stable imp7 KD cells only at earlier time points and the effect was specific because back complementation of imp7 rescued the phenotype. Moreover, the lower level of HIV-1 infection in imp7 KD cells could not be simply explained by cell toxicity or other off-site effects because SIVmac and HIV-2 were not affected. The different behaviour of HIV-1 and HIV-2 or SIVmac in imp7 KD cells was surprising and raised the possibility that these viruses may use distinct pathways for nuclear trafficking.

Imp7 has been shown to bind to HIV-1 IN [20,37] and mutations that abolish this interaction were shown to inhibit both reverse transcription and nuclear entry [37]. However, because of the pleiotropic effects that IN mutations have on virus replication, it was not possible to firmly conclude that the imp7-IN interaction was responsible for the observed phenotype [37]. We found that HIV-2 and SIVmac infection was not significantly impaired in imp7 KD cells and this prompted us to test if their INs were able to bind to imp7. Our pull down interaction assays confirmed that imp7 does indeed specifically bind HIV-1 IN. Side-by-side comparisons showed that the interaction is significantly weaker with HIV-2 and SIVmac INs, while EIAV IN did not show detectable binding. Remarkably, the apparent binding affinity closely correlated with the infectivity of the lentiviruses on imp7 KD cells, strongly suggesting that the imp7-IN interaction is critical for imp7 function. Overall the available evidence points to the existence of varied and possibly redundant pathways for nuclear import of lentiviruses.

To test if imp7 KD inhibited RTC nuclear transport, we performed fractionation assays to separate nuclei from cytoplasm following MLV and HIV-1 vector co-infection and analysed viral DNA distribution by Taqman PCR. These studies demonstrated that HIV-1 DNA associates with the nuclei of infected cells with remarkable speed and efficiency in both cycling and cell cycle-arrested cells. Indeed, at 24 h post infection 90–95% of HIV-1 DNA was found in the nuclear fraction. In contrast, nuclear accumulation of MLV DNA was slower in cycling cells and was further inhibited in cell cycle-arrested cells. Importantly, imp7 KD appeared to inhibit nuclear accumulation of HIV-1 DNA.

These results were confirmed in a subsequent set of experiments in which HIV-1 infection and nuclear import (by means of 2LTR detection) were followed over time. HIV-1 infection was reduced in imp7 KD cells compared to DxR cells at 24 h post-infection and the difference was statistically significant. However the reduction was more modest at later time points and was not statistically significant. Viral DNA copy number gradually dropped in time in both cell types, presumably due to dilution and degradation of un-integrated DNA forms. However, 2LTRs circular viral DNA copy number was lower in imp7 KD cells compared to DxR cells. Inhibition of infection was maximal at 24 h post-infection in imp7 KD cells but the difference in 2LTRs viral DNA levels between imp7 KD and control cells was maximal at 48 h. It is possible that accumulation of 2LTRs viral DNA takes longer than integration and viral gene expression, which would explain the time lag. It is also possible that accumulation of 2LTR DNA forms requires a threshold of viral DNA in the nucleus. Together the results of these two sets of experiments strongly suggest that imp7 KD perturbs the kinetics of HIV-1 DNA trafficking to the nucleus. These results reconcile apparently discordant observations on the effect of imp7 KD in HIV-1 infection. In one study where no difference was found between imp7 KD and control cells, FACS analyses were performed 96 h after HIV-1 infection [36], too late a time point.

Our experimental approach, based on stable imp7 KD cells, has certain limitations. Imp7 is one of the most abundant nuclear import proteins, reaching a concentration of approximately 3 μM in HeLa cells [28]. Hence even an effective and stable KD would presumably leave enough imp7 to support essential cell functions. Residual imp7 in the KD cells may at least in part explain the relatively modest inhibition of HIV-1 infection. It should be noted that a similarly modest inhibition of HIV-1 infection was also observed in HeLaP4 cells with a stable KD of LEGDF/p75 [55], by now an established co-factor for virus integration (reviewed in [46]). However, imp7 may not be essential for HIV-1 infection and additional host factors most likely participate in RTC nuclear import [21,23,24].

We hypothesized [20] that imp7 could mediate nuclear import of DNA by facilitating passage of the charged and hydrophilic molecule through the central channel of the nuclear pore complex, which appears to act as hydrophobic and size-selective phase [56-58]. A corollary to this hypothesis is that reverse transcription of the viral genome may be necessary for nuclear import. This was tested using an HIV-1 mutant unable to reverse transcribe [51,52]. This mutant virus buds normally and can effectively saturate TRIM5α_rh_-mediated restriction, indicating that it has no major post-entry defect [38]. A cell fractionation procedure was devised to reduce contamination of nuclei to minimal levels and to eliminate material associated with the external layer of the nuclear envelope. We were also careful to isolate intact nuclei by sedimentation in a sucrose cushion before extracting nucleic acids. A significant amount of viral RNA was found in the nuclei shortly after infection. Although we cannot exclude that some viral RNA was still bound to the external nuclear membrane, the fairly large quantity of viral RNA and the undetectable levels of cyclophilin A mRNA in the nuclear fraction suggested that the signal represented bona fide nuclear import.

Our results are consistent with previous studies that detected rapid nuclear accumulation of Visna virus, HIV-1 and avian sarcoma and leucosis viruses RNA genomes [59-62]. Presently it is unclear if nuclear import of HIV-1 RNA has any physiological significance. Rapid trafficking of viral RNA into the nucleus also suggests that HIV-1 uncoating may take place before completion of reverse transcription.

Importantly, imp7 KD reduced nuclear accumulation of viral DNA but not viral RNA. Consistent with the possibility that imp7 primarily facilitates translocation of DNA across the nuclear pore complex, plasmid DNA transfection efficiency was also reduced in imp7 KD cells. The defect was specific because it was reversed upon imp7 back-complementation and was sequence independent. Cell cycle-arrest clearly inhibited DNA transfection but imp7 KD had an additive effect. Taken together these experiments suggested that HIV-1 might exploit imp7 to maximise the nuclear import rate of its DNA genome.

Based on our results, we propose a model whereby nuclear import factors need to be specifically recruited onto the viral DNA within the HIV-1 RTC. Thus HIV-1 has evolved the ability to recruit imp7 via IN. This promotes trafficking of the viral DNA across the nuclear pore complex by modifying the overall charge of the nucleoprotein complex. Lentiviruses other than HIV-1 must have evolved the ability to recruit alternative import factors that have properties similar to imp7 and can also promote DNA and/or RNA nuclear import. It will be interesting to know which factor(s) substitute for imp7 in the other lentiviruses.

Why has HIV-1 evolved a very efficient and rapid nuclear import mechanism and what could be the physiological outcome of delaying this process? Our data showed that imp7 KD reduced HIV-1 infection equally in cycling or cell cycle-arrested cells. This suggests that normally HIV-1 does not enter the nucleus via two independent pathways, one for cells in interphase and another for mitotic cells, in agreement with previous studies [21,63-65]. Thus, HIV-1 may use the same mechanism to enter the nucleus of non-dividing as well as dividing cells, such as activated CD4+ T-lymphocytes. Delayed nuclear import may be almost inconsequential in long-lived cells, like terminally differentiated macrophages, where the RTC would have sufficient time to reach the nucleus before the infected cell dies. In contrast, rapid import may be critical in short-lived cells, such as activated CD4+ T-cells [66-68], where a 24-h delay may make the difference between productive and aborted infection. Because activated CD4+ T cells are the main target for HIV-1 infection in vivo [66], we propose that, even if not essential for infection, host cell factors contributing to maximise the rate of HIV-1 infection may play a role in AIDS pathogenesis.

## Materials and methods

### Cell culture and virus production

HeLa and 293 T cells were grown in Dulbecco's modified Eagle's medium (DMEM) (Gibco Labs, Paisley, UK) supplemented with 10% foetal calf serum (FCS) (Helena Bioscience, Newcastle, UK) and 2 mM glutamine at 37°C in 5% CO_2_. Jurkat cells were grown in RPMI medium supplemented with 10% FCS at 37°C in 10% CO_2_. HIV-1 vectors were made and purified as described previously [48] using plasmids pHR' or pCSGW expressing GFP, pCMVΔR8.2, (expressing gag-pol) or pHIV_LAI_Denv GFP and pMD.G, expressing VSV-G [38,39,4]. MLV, SIVmac, HIV-2 and EIAV vectors [40,42,69,70] were prepared and purified using the same procedure. Reverse transcriptase (RT) activity was measured by the Lenti-RT™ Activity Assay (Cavidi Tech, Uppsala, Sweden) following the manufacturer's instructions. HIV-1 p24 was quantified by ELISA using the Coulter Antigen detection assay (Coulter Corp., Miami, FL).

### Knock down of imp7

Synthetic oligonucleotides 5'-ATGGAGCTCTGTATATGGTTGGTTCGCCAATCATATGCAGGGCTCCATCTTTTT-3' and 5'-CTAGAAAAAGATGGAGCCCTGCATATGATTGGCGAACCAACCATATACAGAGCTCCAT-3' designed according to siRNA sequences for imp7 (20) and oligonucleotides 5'-TAATGCAGAAGAAGACCATGGGTTCGCCCATGGTCTTCTTCTGCATTACTTTTT-3' and 5'-CTAGAAAAAGTAATGCAGAAGAAGACCATGGGCGAACCCATGGTCTTCTTCTGCATTA-3' designed to target DsRed were annealed and cloned into U6 promoter driven HIV-1 vectors as previously described [38]. Infected HeLa cells were selected in medium containing 1.5 micrograms/ml puromycin for 1 week; infected Jurkat cells were selected in medium containing 12 micrograms/ml puromycin for one week followed by selection in 6 micrograms/ml puromycin for a further week. After selection cells were frozen in small aliquots in N_2 _or used within 4 passages. Two silent mutations were introduced into the human importin 7 cDNA (Origene, Rockville, MD) using the QuickChange II XL site directed mutagenesis kit (Stratagene, La Jolla, CA) with primer CCTCGAAAAAAAGATGG**T**GCCCTGCA**C**ATGATTGGC (mutations in bold). The plasmid was co-transfected with a Hygro^r ^pUC plasmid into HeLa imp7 KD cells and cells selected in media containing 500 micrograms/ml hygromycin B and 1 microgram/ml puromycin. For infections, HeLa cells were plated onto 24 well plates 4 × 10^4^/well for sh-imp7 cells and 3 × 10^4^/well for sh-DxR cells. Cells were infected 24 hours later with 3-fold serial dilutions of viral stocks and analyzed by FACS 24–72 hours after infection. Jurkat cells were seeded into 96 well plates at 6 × 10^4^/well (sh-imp7 cells) and 4 × 10^4^/well (sh-DxR cells), infected with 3 fold serial dilutions of viral stock and analysed by FACS 20 to 24 hours after infection.

### PCR

PCR was performed in a final volume of 50 microliters containing 1× PCR buffer, 100 μM each dNTP, 2.5 mM MgCl2, 5 U Taq polymerase (Promega) and 30 picomoles of each primer. Primer sequences were as follows: cyclophilin A (exon 1) forward 5'-ATCGAGAATTCCCACCATGGTCAACCCCATCGTGTT-3' and cyclophilin A (exon 5) reverse complementary 5'-TCGATTTCGAATTAGATTTGTCCACAGTCAGCAAT-3' Cycle parameter were: 94°C for 3 min the first cycle, 94°C for 1 min, 550C for 1 min, 680C for 1 min for 27–35 cycles.

### Cell cycle arrest

G1/S cell-cycle arrest was induced by addition of 1.5 micrograms/ml aphidicolin (Sigma, St. Louis, MO) for 24 h in standard medium. Cells were resuspended in 50 microliters PBS+3% FCS and fixed by addition of 1 ml cold 80% ethanol and incubation at 4°C for 30 minutes. Cells were resuspended in 0.5 ml PBS supplemented with 0.25% NP-40 (IGEPAL CA-630) (Sigma) and 2 U of DNAse-free RNAse A (Roche), incubated for 30 mins at 37°C and centrifuged. The pellet was resuspended in 400 microliters PBS containing 0.2 micrograms/ml Propidium iodide (Sigma) and analyzed by FACS. Cell viability was analyzed with the LIVE/DEAD kit (Molecular Probes) following the manufacturers instructions.

### Cell fractionation and detection of viral RNA and DNA

Approximately 5 × 10^6 ^HeLa cells were plated onto 175 cm^2 ^flasks. The next day cells were infected at an MOI of 0.2, incubated 2 hours at 4°C and transferred at 37°C for 4–24 hours. Cells were trypsinized, washed once in PBSA and the pellet was resuspended in 0.5 ml isotonic buffer 1 (20 mM HEPES pH 7.4, 110 mM KCl, 5 mM MgCl_2_, 0.5 mM EGTA, 1 mM DTT, 20 μg/ml aprotinin, 20 micrograms/ml leupeptin). Samples were centrifuged for 2 min at 2,000 rpm at 4°C in a benchtop centrifuge; the pellet was gently resuspended on ice in 50 microliters isotonic buffer 1, then 0.5 ml isotonic buffer + 0.5% NP-40 was added and samples were incubated for 5–7 mins on ice. Following a 2 min centrifugation as before the supernatant was cleared by centrifugation at 9,000 rpm for 20 min at 4°C. The pellet was washed in 0.5 ml isotonic buffer once, resuspended in 1 ml isotonic buffer 2 (50 mM TrisHCl pH 7.5, 25 mM KCl, 5 mM MgCl_2_, 0.25 M sucrose) mixed with 2 ml isotonic buffer 2 + 2.3 M sucrose and placed in a 5 ml ultracentrifuge tube on ice. Samples were underlayed with 1 ml isotonic buffer 2 + 2.3 M sucrose and centrifuged at 36,000 rpm in a Sorvall AH-650 rotor at 4°C for 40 min. The interface containing purified nuclei was collected and nuclei counted by mixing with trypan blue. Nuclei and cytoplasmic extracts were mixed with 1 volume of 2× lysis buffer (100 mM Tris-HCl pH 8, 1% SDS, 10 mM EDTA, 50 micrograms/ml proteinase K), incubated for 4 hours at 55°C and nucleic acids were isolated by phenol/cholorform and ethanol precipitation. Samples were divided into aliquots; one aliquot was treated with RNAse A for 1 hour at 37°C and then subjected to phenol/chloroform extraction and ethanol precipitation. Another aliquot was treated with 2 U/micrograms nucleic acids ReQ1 DNAse in 1× buffer (Promega) for 30 mins at 37°C, then supplemented with 2 mM EGTA to stop the reaction, and samples were incubated at 60°C for 20 min. The Superscript III kit (Invitrogen) was used for first-strand cDNA synthesis primed by random hexamers following the manufacturer's instructions. Control samples were incubated in parallel without RT. TaqMan quantitative PCR was performed using an ABI Prism 7000 thermocycler as described [48]. For amplification of intermediate and late products of reverse transcription, 0.3 μM of each GFP or HIV late primers [48], 0.15 μM of the probe, 12.5 μl of Quantitect Probe Master Mix (Qiagen) were used in a final volume of 25 μl. The standards were prepared with CNCG or pHR' plasmids in 10-fold dilutions from 10^7 ^to 10^2 ^copies/microliter. 200–500 ng of template DNA was added per reaction. For amplification of 2LTR circular DNA, the same conditions were used with primers 2LTRqPCRF: 5'-AACTAGAGATCCCTCAGACCCTTTT-3' and 2LTRqPCRRC: 5'-CTTGTCTTCGTTGGGAGTGAATT-3' and probe 5'-FAM-CTAGAGTTTTCCACACTGAC-0-TAMRA-3'. Standards were prepared by PCR amplification of DNA from acutely infected cells with primers 2LTRF 5'-GCCTCAATAAAGCTTGCCTGG-3' and 2LTRRC 5'-TCCCAGGCTCAGATCTGGTCTAAC-3'. The amplification product was cloned into TOPO vector, amplified and confirmed by sequencing. MLV DNA was amplified using primers MLF-F, 5'-CATGGACACCCAGACCAG-3'; MLV RC 5'-CGGATGGAGGAAGAGGAG-3'; MLV probe, 5'-FAM-CCCCTACATCGTGACCTGGGAAGCC-TAMRA-3' and pCNCG as standard.

### Western blot

Antibody C-20 against Ran was purchased from Santa Cruz Biotechnology (Santa Cruz, CA). The anti-imp 7 antibody was described previously [20]. Anti-rabbit and anti-goat IgG HRP-conjugated antibodies were purchased from Jackson Laboratories (Bar Harbor, MN) or Sigma-Aldrich. After SDS PAGE, the proteins were transferred to PVDF membranes (Bio-Rad, Hercules, CA) and probed with the primary antibodies. HRP-conjugated secondary antibodies were used diluted 1:3,000 in PBS containing 10% non-fat milk. Chemiluminescence (ECL, Amersham) was used to develop the blots as described by the manufacturer. Autoradiography films were exposed for different periods of time to ensure linearity of the signal.

### Transfections

HeLa cells were plated into 24-well plates at 4 × 10^4 ^cells/well or in 6-well plates at 3 × 10^5 ^cells/well and transfected the next day. To transfect cells in 24-well plates, 10 microliters linearized plasmid (70 ng/μl in TE) were mixed with a solution of 50 μl Opti-Mem medium (Invitrogen) and 4.6 μl Fugene-6 (Roche Molecular Biochemicals), incubated for 15 mins at room temperature and then 0.5, 1, 3, 9 and 18 μl/well of the solution were added to the cells after media change to 0.5 μl fresh DMEM + 10% FCS. To transfect cells in 6-well plates all doses were scaled up by a factor of four. Cells were analyzed by FACS 24 hours after transfection to detect GFP expression.

### Recombinant proteins and pull-down experiments

To make pGEX6P-imp7, used for expression GST-tagged Imp7, a BamHI/HindIII fragment of pQE9-imp7 [29], spanning the imp7 coding region, was subcloned between BamHI and SmaI sites of pGEX6P3 (GE Healthcare) (the HindIII DNA end was filled in using T4 DNA polymerase to allow blunt-end ligation). Escherichia coli Rosetta 2 cells (Novagen) transformed with pGEX6P-imp7 were grown in shake flasks in Lennox LB medium at 30°C to an A_600 _of 0.9–1.0 before addition of 1% glycerol, 2.5% ethanol, and 0.25 mM IPTG. Following 5-h induction at 15°C, the cells were harvested and stored at -80°C. To isolate GST-imp7, thawed bacterial paste was lysed by sonication in core buffer (250 mM NaCl, 10% (v/v) glycerol, 50 mM Tris-HCl, pH 7.4), supplemented with 10 mM DTT, 2 mM EDTA, and 0.5 mM PMSF. Cell debris were removed by centrifugation, and GST-imp7 was captured on glutathione sepharose (GE Healthcare). The protein was eluted in core buffer supplemented with 50 mM reduced glutathione or incubated with human rhinovirus 14 3C protease to release untagged imp7. The proteins were further purified by size exclusion chromatography on a preparative Superdex 200 column (GE Healthcare) operated in 250 mM NaCl, 50 mM Tris-HCl, pH 7.4, concentrated using a Centriprep-YM3 device (Millipore), supplemented with 10 mM DTT and 10% glycerol and snap-frozen in liquid nitrogen. C-terminally His_6_-tagged and non-tagged IN proteins were prepared as previously described [43,71,72].

Pull down assays were done according to published procedures [43,73,74] with slight modifications. For GST pull-downs, 10 micrograms BSA and 10 micrograms untagged IN protein were added to ice-cold suspension of 15 μl glutathione sepharose (settled resin volume) carrying immobilized GST or a GST fusion protein (2 mg/ml) in 800 μl pull down buffer 1 (PDB1: 130 mM NaCl, 2 mM MgCl_2_, 2 mM DTT, 0.1% NP-40, 50 mM Bis-Tris propane-HCl, pH 7.45). The samples were gently rocked for 2.5 h at 4°C and washed in three changes of ice-cold PDB1, allowing the beads to precipitate on ice after each wash. The proteins eluted by boiling in Laemmli SDS sample buffer were resolved in tris-glycine 11% SDS PAGE gels. Concentration of NaCl in PDB1 was adjusted as explained in the results section. For His6-tag pull down assays, 10 ug of each BSA, Imp-7, and a C-terminally His_6_-tagged IN protein were incubated with 20 ul Ni-NTA agarose (Qiagen) suspended in 800 ul of pull down buffer 2 (PDB2: 150 mM NaCl, 2 mM MgCl_2_, 35 mM imidazole, 0.1% NP-40, 50 mM Bis-Tris propane-HCl, pH 7.45) for 5 h at 4°C with gentle rocking. The beads washed in three changes of ice cold PDB2 were boiled in Laemmli SDS sample buffer supplemented with 50 mM EDTA, and the eluted proteins were resolved in tricine 10–20% SDS PAGE gels (Invitrogen).

## Abbreviations

IN: Integrase protein; SIV: Simian immunodeficiency virus; EIAV: Equine infectious anemia virus; imp7: importin 7; tnp3: transportin 3; LEDGF: Lens epithelial-derived growth factor; CA: capsid protein.

## Competing interests

The authors declare that they have no competing interests.

## Authors' contributions

LZ designed and performed experiments, analyzed data and contributed to writing the paper, PC performed experiments (IN pull-down assays), analyzed data and contributed to writing the paper, LL performed experiments and analyzed data shown in Fig. 5, SW and JR contributed the imp7 shRNA plasmid construct, AF designed and performed experiments, analyzed data and wrote the paper. All authors have read and approve the manuscript.

## Supplementary Material

Additional File 1 **Figure S1.** Growth kinetics of HeLa DxR, imp7 KD cells and imp7 back-complemented cells re-expressing imp7 (7+7R). (B) Growth kinetics of Jurkat DxR and imp7 KD cells. Click here for file

Additional File 2 **Figure S2**. Inhibition of HIV-1 infection in imp7 KD cells is dependent on the MOI and on the virus producer cells. (A) DxR KD (shDxR), imp7 KD (shimp7) HeLa cells were plated onto 24-well plates to obtain equal cell densities by the following day, when they were infected with five-fold serial dilutions of a VSV-G pseudotyped HIV-1 vector (pHR') expressing GFP at MOIs ranging from 0.01 to 1.25. Twenty-four hours after infection the percentage of GFP+ cells was measured by flow cytometry. Data are representative of two independent experiments. (B) The same VSV-G pseudotyped HIV-1 vector expressing GFP was produced in DxR KD (DxR virus) or in imp7 KD (imp7 virus) HeLa cells. Virus stocks were normalized for RT activity and used to infect DxR KD or imp7 KD HeLa cells at an MOI of 0.05. Twenty-four hours after infection GFP+ cells were measured by flow cytometry. Data are shown as average percentage of infected (GFP+) cells relative to DxR KD control ± SD of three independent experiments using two different viral stocks. Click here for file
