# Supplementary figures and images for: HIV-1 exploits importin 7 to maximize nuclear import of its DNA genome
Source: Retrovirology. 2009 Feb 4;6:11. doi: 10.1186/1742-4690-6-11 (PMC2660290; doi:10.1186/1742-4690-6-11)

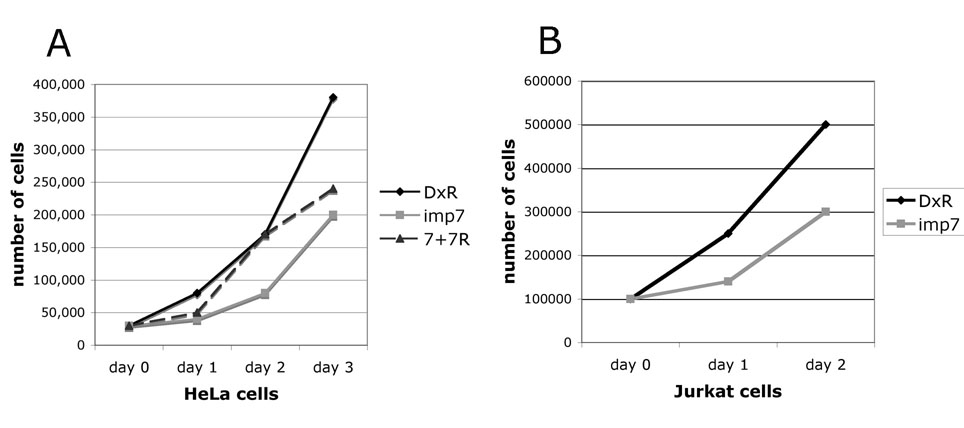

Supplement: Additional File 1 — Figure S1. Growth kinetics of HeLa DxR, imp7 KD cells and imp7 back-complemented cells re-expressing imp7 (7+7R). (B) Growth kinetics of Jurkat DxR and imp7 KD cells. [file 1742-4690-6-11-S1.jpeg]

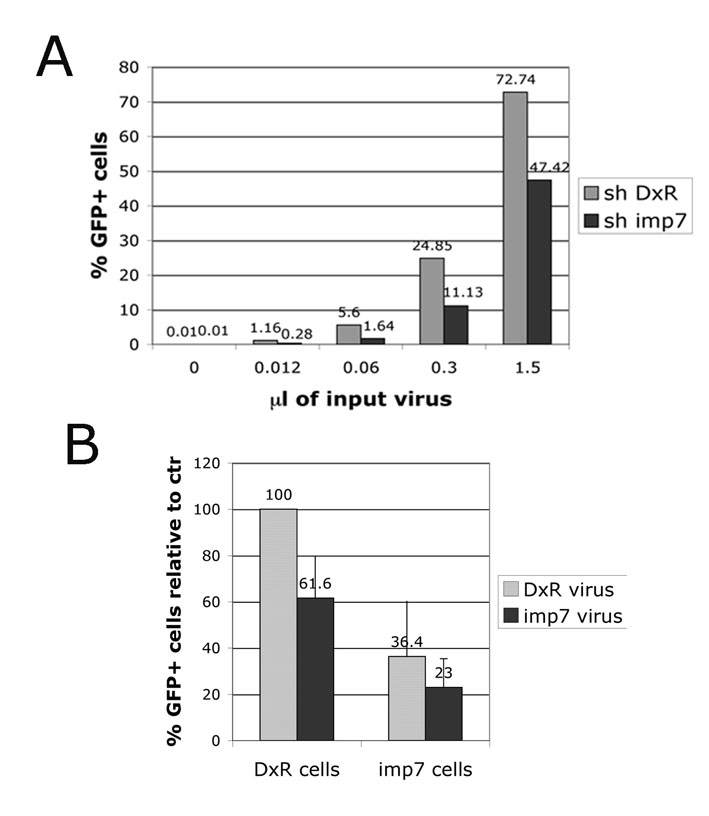

Supplement: Additional File 2 — Figure S2. Inhibition of HIV-1 infection in imp7 KD cells is dependent on the MOI and on the virus producer cells. (A) DxR KD (shDxR), imp7 KD (shimp7) HeLa cells were plated onto 24-well plates to obtain equal cell densities by the following day, when they were infected with five-fold serial dilutions of a VSV-G pseudotyped HIV-1 vector (pHR') expressing GFP at MOIs ranging from 0.01 to 1.25. Twenty-four hours after infection the percentage of GFP+ cells was measured by flow cytometry. Data are representative of two independent experiments. (B) The same VSV-G pseudotyped HIV-1 vector expressing GFP was produced in DxR KD (DxR virus) or in imp7 KD (imp7 virus) HeLa cells. Virus stocks were normalized for RT activity and used to infect DxR KD or imp7 KD HeLa cells at an MOI of 0.05. Twenty-four hours after infection GFP+ cells were measured by flow cytometry. Data are shown as average percentage of infected (GFP+) cells relative to DxR KD control ± SD of three independent experiments using two different viral stocks. [file 1742-4690-6-11-S2.jpeg]
